# LV rotational mechanics in patients with dilated cardiomyopathy compared to healthy individuals: Experience from the European CMR Registry

**DOI:** 10.1186/1532-429X-17-S1-Q69

**Published:** 2015-02-03

**Authors:** Andreas Ochs, Andreas Schuster, Johannes Riffel, Jan Düchting, Simeon Thome, Florian Andre, Sebastian A Seitz, Christian Galuschky, Heiko Mahrholdt, Oliver Bruder, Grigorios Korosoglou, Hugo A Katus, Sebastian Buss

**Affiliations:** 1 Department of Cardiology, University of Heidelberg, Heidelberg, Germany; 2 Department of Cardiology and Pneumology and German Centre of Cardiovascular Research (DZHK Partner Site), Georg-August-University, Göttingen, Germany; 3 TomTec Imaging Systems GmbH, Unterschleissheim, Germany; 4 Department of Cardiology, Robert Bosch Medical Center, Stuttgart, Germany; 5 Department of Cardiology and Angiology, Contilia Heart and Vascular Center, Essen, Germany

## Background

Left ventricular rotation is an important part of myocardial mechanics during the cardiac cycle. Understanding the mechanisms of LV rotation in different cardiac diseases could play an important role for diagnosis, risk stratification and prediction of heart failure. We sought to analyze LV rotation using the feature tracking technique in patients with dilated cardiomyopathy (DCM) included in the European CMR Registry.

## Methods

82 Patients diagnosed with DCM and a control group consisting of 30 healthy volunteers were analyzed using dedicated feature tracking imaging software (2D CPA MR©, TomTec Imaging Systems GmbH). An apical, midventricular and basal slice in short axis orientation were tracked to analyze the peak rotation of each slice. LV twist (defined as the difference of peak apical and peak basal rotation at isochronal time points) and LV torsion (defined as the LV twist per ventricular length) were calculated.

## Results

DCM patients and controls with normally directed rotation (counterclockwise apical and clockwise basal rotation) were compared to each other (table 1): DCM patients showed significantly lower apical and basal rotation resulting in significantly lower LV twist and LV torsion.

**Table 1:** Comparison of DCM patients and the control group with normally directed rotation

	n	apical rotation (°)	basal rotation (°)	twist (°)	torsion (°/cm)
DCM	47	4.06±2.55	-4.18±2.17	7.36±3.87	1.36±0.73

control group	24	5.54±1.76	-5.58±2.15	9.98±2.91	2.31±0.82

significance		p<0.02	p<0.02	p<0.01	p<0.001

Amongst the DCM patients an inversed rotational pattern was frequently observed (Table 2): 57.3% of DCM patients showed a normal direction of rotation, compared to 80% of the control group. 30.5% of patients with DCM and 3.3% of the control group showed inversed clockwise apical rotation. 16.7% of the healthy volunteers showed an inversed counterclockwise rotation of the basis compared to 9.8% of patients with DCM. An inversed rotation in opposite directions of both, the apical and the basal layer, was present in 2.4% of DCM patients, but not in control subjects.

**Table 2:** Direction of rotation: Distribution of DCM patients and the control group

	n	mean age	normal rotation	inversed apical rotation	inversed basal rotation	inversed rotation in both layers
DCM	82	58.0±14.0	47 (57.3%)	25 (30.5%)	8 (9.8%)	2 (2.4%)

control group	30	50.5±12.5	24 (80.0%)	1 (3.3%)	5 (16.7%)	0 (0%)

## Conclusions

DCM is associated with an inverted direction of rotation in a significant amount of cases, predominantly affecting the LV apex. Patients with a normal direction of rotation exhibit significant lower torsion. These findings warrant further investigation including clinical follow-up data in order to analyze their impact on clinical outcome in patients with DCM.

## Funding

N/A.

